# Direct Observation of Flat Bands in Near-Magic-Angle Twisted Bilayer CVD Graphene

**DOI:** 10.1021/acs.nanolett.6c01400

**Published:** 2026-07-27

**Authors:** Gianluigi Baiardi, Alex Boschi, Giulia Piccinini, Vaidotas Mišeikis, Lorenzo Cavicchi, Aaron Bostwick, Chris Jozwiak, Eli Rotenberg, Kenji Watanabe, Takashi Taniguchi, Marco Polini, Fabio Beltram, Antonio Rossi, Stiven Forti, Sergio Pezzini, Camilla Coletti

**Affiliations:** † Center for Nanotechnology Innovation @NEST, 121451 Istituto Italiano di Tecnologia, P.za San Silvestro 12, Pisa, PI 56127, Italy; ‡ NEST Laboratory, 19004 Scuola Normale Superiore, P.za San Silvestro 12, Pisa, PI 56127, Italy; § Scuola Normale Superiore, P.za dei Cavalieri 7, Pisa, PI 56126, Italy; ∥ Advanced Light Source, Lawrence Berkeley National Laboratory, 6 Cyclotron Rd, Berkeley, California 94720, United States; ⊥ Research Center for Electronic and Optical Materials, 52747 National Institute for Materials Science, 1-1 Namiki, Tsukuba, Ibaraki 305-0044, Japan; ▽ Research Center for Materials Nanoarchitectonics, National Institute for Materials Science, 1-1 Namiki, Tsukuba, Ibaraki 305-0044, Japan; 7 Dipartimento di Fisica dell'Università di Pisa, Largo Bruno Pontecorvo 3, I-56127 Pisa, Italy; 8 Istituto Nanoscienze - CNR, NEST-SNS, Piazza San Silvestro 12, Pisa, PI 56127, Italy

**Keywords:** Magic-angle twisted bilayer graphene, Chemical vapor deposition, Nano-ARPES, Flat bands

## Abstract

Advances in chemical vapor deposition (CVD) growth have driven graphene crystal quality to unprecedented levels, yet it is still unknown whether this route can realize the fragile flat-band and correlated states of the magic-angle (MA) twisted bilayer graphene (TBG). Here, we report on the experimental observation by room-temperature nano-angle-resolved photoemission spectroscopy (nano-ARPES) of flat bands in a TBG sample close to the MA, assembled via a grow-and-stack protocol based on low-pressure CVD of graphene on copper. Our study indicates electronic bands fully comparable to those measured in exfoliation-based samples and determines the size of the largest near-MA domain to be compatible with electronic transport experiments, motivating further experiments on flat-band physics in CVD-graphene.

## Graphical abstract

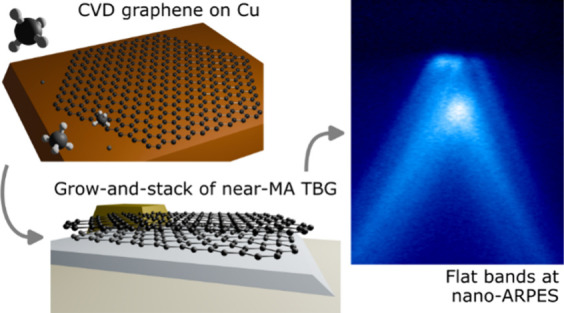

Two-dimensional materials provide a versatile platform for exploring and exploiting novel electronic,^1–3^ optical,^4–7^ and quantum^8–10^ phenomena. When stacked with a relative twist, they form moiré superlattices that host emergent correlated states, flat bands, and exotic phases of matter.^11^ Landmark discoveries, such as superconductivity^12^ and correlated insulators^13,14^ in magic-angle twisted bilayer graphene (MATBG), have been made exclusively with exfoliated flakes. Indeed, mechanical exfoliation represents the method of choice,^15–23^ mostly thanks to the extremely low defect density of the individual layers and the use of clean, dry van der Waals assembly techniques.^24^ However, this reliance on exfoliated samples poses intrinsic limitations in scalability, reproducibility,^25^ and device integration.^26,27^ Growth approaches to 2D crystal production, such as chemical vapor deposition (CVD),^28–31^ offer an alternative that is getting on par in terms of material quality^32–36^ and promises to achieve reliable protocols for the deployment of standardized devices.^37–39^ Having established the capability of producing moiré TBG devices,^40^ a central open question is whether the fragile flat bands at the MA, highly sensitive to charge and twist-angle disorder, can be realized in a scalable platform such as CVD-grown graphene. Here we demonstrate via nano-ARPES the observation of flat bands in twisted bilayer graphene from CVD, establishing a pathway toward scalable moiré materials. In addition, we study the relaxation of the near-MA configuration toward Bernal (AB) stacking across structural defects in the heterostructure, determining the size of the largest domain to be device-compatible for further studies of electronic transport in grown-and-stacked samples.

The key features of the fabricated sample and its geometry for the nano-ARPES experiment are summarized in Figure 1. We selected two CVD-grown monolayer graphene single crystals, each a few hundred micrometers wide and sharing the same crystallographic orientation due to seeded growth on the same Cu grain.^41^ These crystals were used as candidates for the grow-and-stack assembly^40^ of MATBG (Figure 1a). The final structure is schematically depicted in Figure 1b, where the twist angle between the two graphene layers is increased for clarity, making the moiré unit cell (red hexagon) more visible. The pick-and-flip technique described in the Materials and Methods section of the Supporting Information (SI) allowed us to obtain an exposed TBG stack on top of a thin (∼30 nm) hexagonal boron nitride (hBN) flake, ensuring atomic flatness (an optical image of the heterostructure is shown in Figure S1a). The Raman mapping highlights the candidate MA areas (strain soliton contribution to the 2D peak higher than that of Bernal-stacked regions, Figure S1c)^42^ that were subjected to AFM tip-assisted brooming.^43^ After cleaning the sample surface, we performed scanning tunneling microscopy (STM) measurements and observed a clear near-MA moiré pattern, as shown in Figure 1c. The panel reports room-temperature STM topographic imaging of the moiré superlattice, along with a denoised portion obtained using a low-pass filter to enhance its visibility. Bright yellow regions corresponding to local AA stacking are connected by a triangular network of strain solitons (bright blue), similar to the hBN-unaligned MATBG shown in the work by Chen et al.^44^ The moiré lattice parameter determined by the computation of the 2D autocorrelation function evaluates to 12.9 nm, corresponding to a twist angle of about 1.11°.

**Figure 1:**
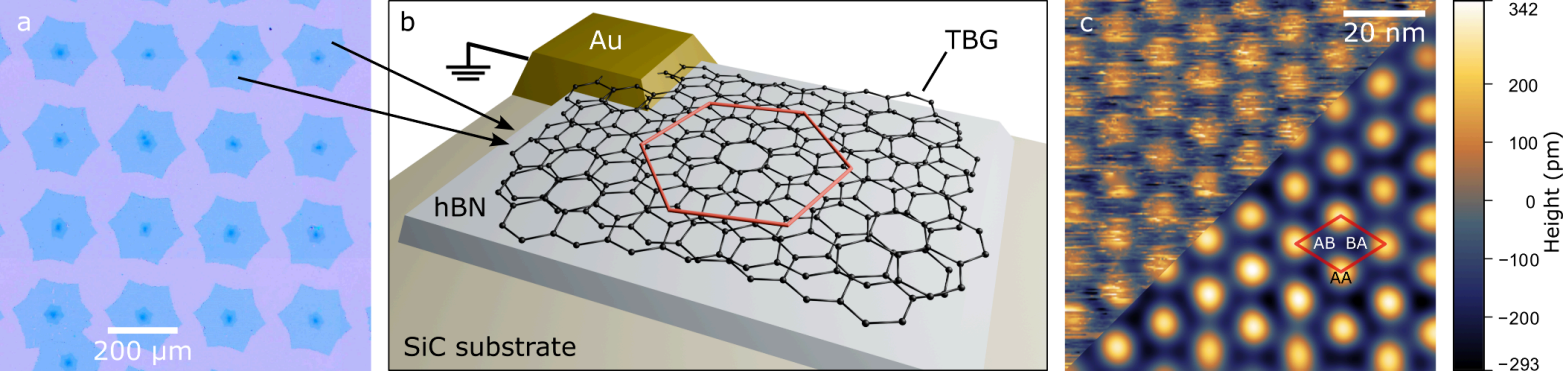
**a.** Graphene crystals previously grown on the same Cu grain and transferred on a Si/SiO_2_ substrate; precise control of the stacking angle is ensured by their mutual alignment. Two of them are highlighted as designed precursors for the fabrication of the near-MATBG device. **b.** The architecture of the assembled device, featuring an exposed TBG structure (the twist angle is exaggerated for readability) on hBN. A red hexagon highlights the moiré superlattice unit cell. **c.** Room-temperature STM imaging of the moiré superlattice, denoised with a low-pass filter in the bottom-right half. A red diamond identifies the primitive cell, containing the two Bernal-stacked regions (labeled in white) and spanning from one AA-stacked spot to another (labeled in black). The STM tip was biased at 50 mV, and the current set point was 0.2 nA.

Once near-MA domain candidates were identified, we performed nano-ARPES at the ALS synchrotron directly on these regions. Full details of the measurements are provided in the Materials and Methods section. Figure 2 reports three cuts across the valence-band photoemission intensity recorded from the largest domain under the ∼1 μm^2^ spot size of the light beam, together with three iso-energy surfaces extracted at different binding energies. Figure 2a displays the ARPES-revealed electronic dispersion of the sample along the direction connecting the closest couple of single-layer Dirac cones, here labeled as *k*_*x*_ and also referred to as the κmκ′ direction in the reference frame of the moiré mini-Brillouin zone. In our spectra, the bands always appear as p-doped, with the shift from the expected neutrality point exceeding 100 meV where the local density of states is lowest, such as in 30°-TBG^45^ reference regions (see Figure S2a). This shift was witnessed also in an analogous sample deposited on a Si/SiO_2_ wafer and could, in principle, result from residual contamination introduced during growth, transfer, or stacking. However, in-house room- and low-temperature transport measurements on multiple devices fabricated with the same protocol all confirm the electrical neutrality of the graphene layers and rule out this scenario. In Figure S2 we compare the carrier concentration in the present sample, as computed with nano-ARPES data, to the one in an exemplary monolayer graphene device, from transport measurements, demonstrating the neutrality of the material when it is not subjected to ionizing radiation. Considering the reported data and analogous ARPES results in other experiments,^46,47^ we attribute the effect to synchrotron-radiation-induced photodoping. We point out that the details of this photodoping effect are not fully clarified; dedicated studies beyond the scope of this work would be required to investigate its dependence on photon flux and possible transient behavior. Regarding the latter, both the sample investigated here and the analogous sample deposited on Si/SiO_2_ exhibited a consistent level of doping throughout the measurement session, with no obvious evolution of the observed band structure. While this observation is compatible with a rapid establishment of the photodoped state under synchrotron illumination, the available data do not allow us to draw firm conclusions on the underlying dynamics. As a consequence of photodoping, the electronic states near the charge-neutrality point were not accessible in our measurements. Nonetheless, by taking a high-symmetry cut slightly off the κmκ′ axis (i.e., along a parallel line, as in Figure 2a) it is possible to observe the onset of the flat bands near the Fermi level: due to the extremely high density of states, the photoinduced carriers reside within a narrow energy window, resulting in partially hole-populated flat bands that are clearly visible (albeit off the K points of graphene). A comparison between the bands observed in our sample along this direction and the standard electronic structure of MATBG computed via the Bistritzer–MacDonald model^48^ is reported in Figure S3, showing overall similarity. The morphology of the signal distribution throughout the whole of Figure 2 is fully comparable to that previously reported in other works for devices close to the MA fabricated from exfoliated graphene.^49,50^ Specifically, the orthogonal cuts along the *k*_*y*_ direction in Figure 2b,c (mγm′ direction) clearly display, from lower to higher binding energy, a flattening of the low-energy band close to the Fermi level and a gap opening resulting from band hybridization at low energies. Moreover, we note that the ARPES intensity from the inner bands is stronger than for the outer ones (Figure 2b), consistently with the spectral weight shift recently reported by Li et al.^51^ for MATBG, adding to the experimental evidence obtained so far in favor of the near-MA configuration. To place our results in the context of previous nano/micro-ARPES studies, we performed a quantitative analysis of representative energy and momentum distribution curves (EDCs and MDCs) extracted from Figure 2b. The resulting flat-band line width and dispersion, gap-related energy scale, and MDC line widths are summarized in Table S1, while the fitting procedures are reported in Figure S4. We point out that the flat bandwidth is not univocally defined in the literature (see Table S1), which also includes substantial differences in experimental conditions among the available studies. Nevertheless, the extracted spectral metrics are broadly consistent with those reported for exfoliated MATBG samples. Isoenergetic intensity distributions are provided in Figure 2d–f for three energy values, illustrating the Fermi surface and the development of an articulated structure derived from the interaction of the Dirac cones close to the MA. Figure 2e,f exhibit an omega-like shape, modulated by the photoemission matrix element, fully compatible with previous studies on near-MA samples.^49^ Overall, the electronic structure measured in reciprocal space indicates that CVD grown-and-stacked graphene can host delicate moiré flat-band configurations, underscoring the high quality achievable with current growth and transfer methods.

**Figure 2:**
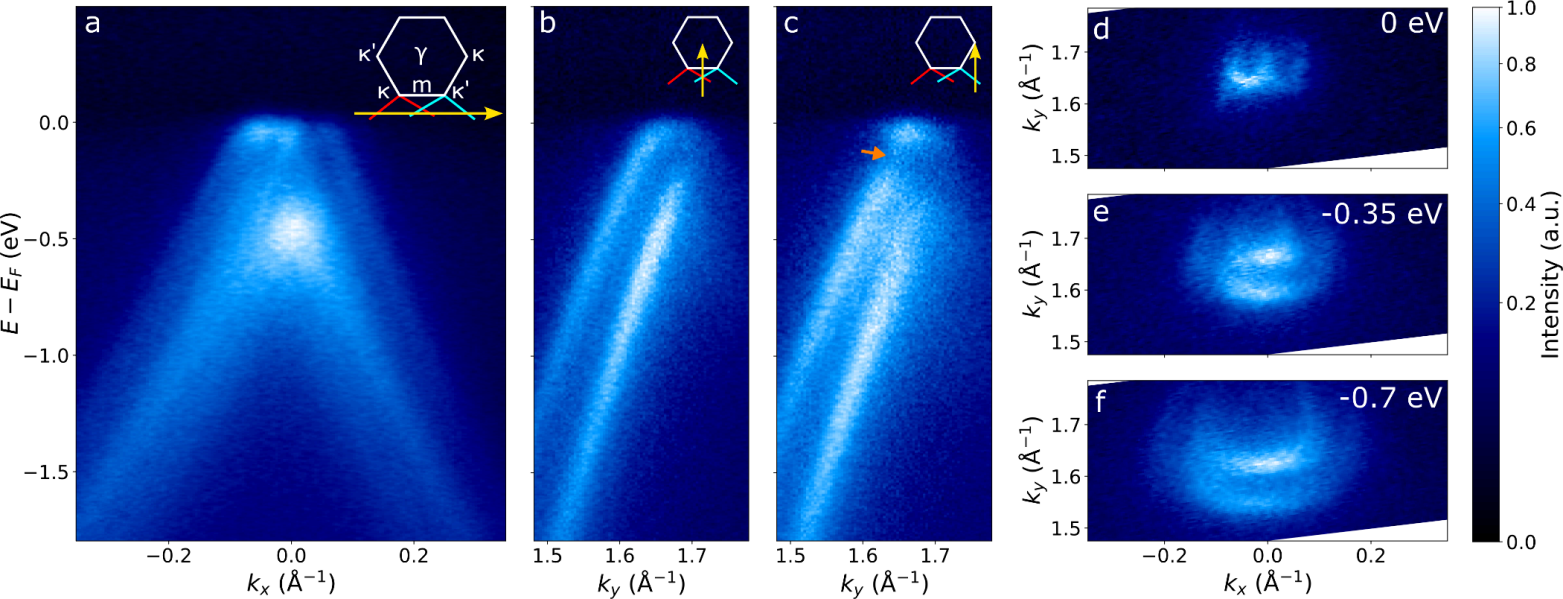
Cuts across the ARPES intensity recorded inside a near-MATBG domain with a photon energy of 27 eV and a pass energy of 30 eV, at room temperature, with a 1 μm^2^ spot size. Coordinates are referred to a single graphene layer Brillouin zone. The insets show a TBG mini-Brillouin zone (white), where the red and blue segmented lines belong to the original first Brillouin zones of the twisted graphene layers. The first inset is enlarged for readability; the rest are on scale with the axes. Notice the nonlinear color scale, mapped to the square root of normalized intensity values to enhance low-intensity features. **a.** Cut along the *k*_*x*_ (κmκ′) direction, slightly off the intersection with the Dirac points of the individual graphene layers. The low-energy hybridization of the original cones allows us to observe the onset of the flat bands, while at higher energies a nontrivial structure featuring multiple bands is present. **b, c.** Cuts along the *k*_*y*_ (mγm′) direction, at *k*_*x*_ values indicated by the yellow arrow in the inset. The flattening of the low-energy band is evident in panel b, while a hybridization-driven gap opens about −0.2 eV in panel c (orange arrow). **d–f.** Isoenergetic cuts at the Fermi level (0 eV), at −0.35 eV, and at −0.7 eV, showing the complex photoemission intensity distribution arising from the hybridization of the Dirac cones, analogous to already reported data in the literature for MATBG devices built out of exfoliated graphene flakes.

Having established the presence of flat bands, we next examine their spatial distribution, as well as the evolution due to relaxation of the twist angle, leveraging the 1 μm^2^ resolution of nano-ARPES. To this end, Figure 3 displays the analysis of a single hyperspectral data set. In Figure 3a, the signal at each location is obtained by integrating the ARPES intensity over a shallow window around the origin of the *k*_*x*_ and *E* axes, covering 0.15 Å^–1^ in momentum and 40 meV in energy (its perimeter is shown as a yellow rectangle in panels b–d). The binning size is enough to capture the full contribution of the flat-band region and distinguish the lower signal provided by the steeper dispersion of Bernal-stacked areas. As a result, locations endowed with near-MA dispersion stand out from the rest and allow initially estimating in 15 × 10 μm^2^ the extension of the near-MA domain under observation. To further strengthen the identification of electronically different domains in the sample, we implemented an unsupervised machine-learning analysis based on Non-negative Matrix Factorization (NMF). NMF decomposes the hyperspectral data set into a small number of non-negative spectral components and their corresponding spatial distributions. Compared to Principal Component Analysis (PCA), NMF is particularly suitable for photoemission data because both the spectral intensity and the extracted components are constrained to be positive, resulting in a more physically interpretable decomposition in terms of distinct electronic contributions. NMF identifies four domains, represented in Figure 3a via solid contour lines, that match the three representative dispersions in Figure 3b–d, plus a fourth region with scarce signal (due to the physical edge of the sample, bottom-right, and to the lack of the TBG structure, top-right). Details on the procedure are reported in the SI. The orange dotted square in Figure 3a highlights the AFM-broomed area, which displays substantially less noise than the surroundings in the ARPES signal acquired. The same panel includes as a red dashed line the position of a wrinkle in the hBN flake which likely occurred during fabrication. The asperity induced by this defect appears to cause the near-MA configuration to fully relax to Bernal stacking at that location and beyond. As is visible in Figure S6, across the wrinkle the overall bilayer orientation on the substrate experiences discontinuities that locally misorient both layers of graphene, leading to their rearrangement in the most favorable configuration regardless of the angle imposed during the assembly. This wrinkle-induced relaxation highlights a residual degree of uncertainty in the outcome of current state-of-the-art dry assembly techniques, which can be mitigated only up to a certain extent.^15,52^ To increase the MATBG yield and domain size, one could devise new stacking schemes that reduce as much as possible the human factor,^53^ as well as the mechanical stress on the crystals (see for instance the low-pressure technique recently introduced to preserve rhombohedral stacking^54^).

**Figure 3:**
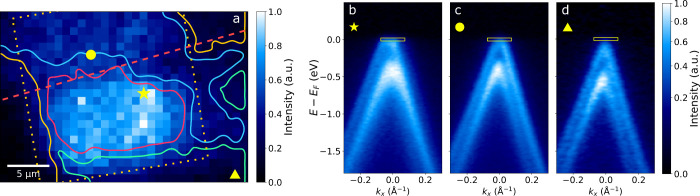
**a.** Real-space imaging of the largest near-MA domain in the sample, highlighted by integrating the room-temperature ARPES intensity on a 0.15 Å^–1^-wide and 40 meV-deep window about *k*_*x*_ = 0 and the Fermi level, displayed in yellow in panels b–d. The spatial distribution of the spectral components identified by NMF applied to the nano-ARPES hyperspectral data set is displayed with solid-line contours at 80% assignment probability obtained via a Gaussian Mixture Model. The analysis separates a near-MA region (red contour), a Bernal-stacked region within the AFM-broomed area (cyan contour), and a Bernal-stacked region outside the broomed area (green contour). The orange dotted square marks the AFM-broomed area, while the red dashed line indicates the position of a wrinkle in the underlying hBN flake. **b–d.** Representative κmκ′-parallel ARPES cuts acquired at the positions marked in panel (a), corresponding to the near-MA region (b), the broomed Bernal region (c), and the nonbroomed Bernal region (d). The latter exhibits a shifted apparent Fermi level, consistent with a stronger influence of residual surface contamination. All cuts are acquired off the K points of both graphene layers as in Figure 2. The color scale is identical to that of Figure 2. Data were acquired with a photon energy of 95 eV and a pass energy of 50 eV.

To obtain a more quantitative estimation of the length scale over which the MA configuration of this large domain relaxes, an approach based on fitting of position-resolved EDCs was adopted (Figure 4). This method allows pinpointing the evolution of the low-energy band by building a more robust indicator of its shape than the simple integrated intensity as in Figure 3. Initially, binned EDCs have been extracted across the near-MA domain along its short side (see Figure S6a for the exact direction of the cut). The binning in *k*_*x*_ was set again to 0.15 Å^–1^ around the center of the *k*_*x*_ axis (Figure S6b), to concentrate the spectral weight of the flat band into a narrow energy range, while diluting contributions from Bernal-like and higher-energy bands. In this way, flatter dispersions manifest as sharper peaks in the binned EDCs (Figure S6c). Figure 4a displays the first series of position-resolved EDCs, extracted along the white arrow in Figure S6a, with the color-mapped ARPES intensity separately normalized within each EDC. The onset of the flat band is clearly visible in the raw data in the central region of the plot (yellow arrow), owing to their strong intensity. However, to take into account Fermi distribution effects, the next step is to fit each EDC as exemplified in Figure 4b,c for two opposite cases, corresponding to a Bernal-relaxed location out of the near-MA domain and a site inside the domain, respectively. The procedure includes a prior Shirley background subtraction and the fitting of two unconstrained components, built as a product of a Lorentzian profile and the Fermi distribution, subsequently convolved with a Gaussian distribution to include instrumental uncertainty (see SI for details). The Lorentzian profile center position of the weaker component (in blue in Figure 4b,c) for each EDC is displayed in Figure 4d and constitutes the desired indicator of the electronic configuration shape across the near-MA domain: in the middle of the domain, the flat band yields a photoemission intensity concentrated just below the Fermi level throughout the *k*_*x*_-binning range and thus assigned by the fitting algorithm to a component centered at the Fermi level; conversely, where the low-energy band is more dispersive, the center position of the associated component is shifted in energy to more negative values, eventually saturating for Bernal-stacked areas where the dispersion is the steepest. To improve consistency, Figure 4d aggregates the results of five parallel series of EDCs (the remaining four are reported in Figure S7, their directions being the green-shaded arrows in Figure S6a) and thus displays the average position of the low-energy band, with ±σ as the shaded area. We note that the method performs better inside the less noisy and doped AFM-broomed region (i.e., up to ∼17 μm), while out of it the curve does not seem to reach a stable value. Considering the apparent bell-like decay of the magic-angle configuration, its relaxation length can be described as half fwhm, equal to 4.1 ± 1.1 μm. With this analysis being done on the shorter side of the domain, this result marks an essential achievement in view of fabricating MATBG devices starting from graphene synthesized by a scalable technique such as CVD. By performing the same procedure along the other axis of the domain, it is possible to give a new estimation of the domain size as 84 ± 41 μm^2^. The high uncertainty is due both to the measurement resolution, relatively low with respect to the size of the near-MA domain, and to limitations of the method itself, which possibly trades fine details of the electronic dispersion for an improved signal. Nevertheless, this surface extension represents a promising scale for studying the homogeneity of the electronic properties^55^ in electrical transport experiments.

**Figure 4:**
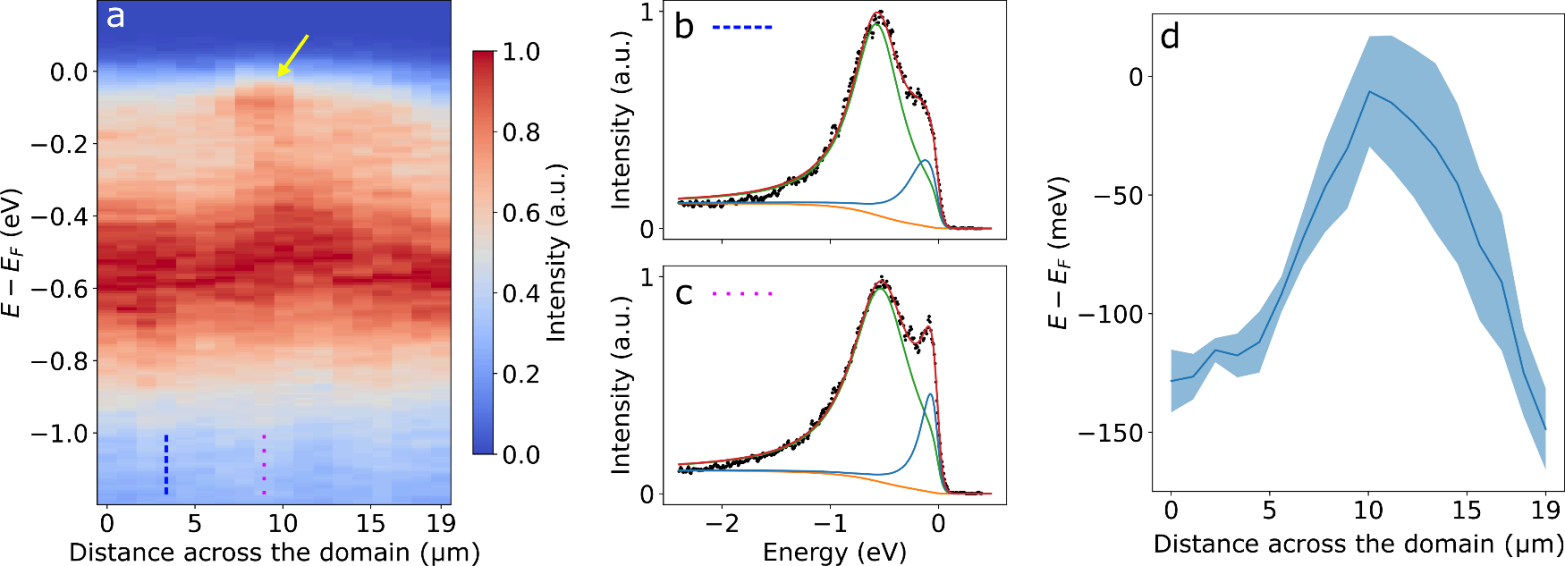
Estimation of the magic-angle configuration relaxation length. **a.** *k*_*x*_-binned EDCs extracted across the near-MA domain (along the white arrow in Figure S6a). The largest contribution extending about −0.5 eV in each EDC is due to the integrated signal of the remote band. A weaker signal, due to the low-energy band, is responsible for the still relevant intensity close to the Fermi level, in few cases standing out as a prominent peak (marked by the yellow arrow). **b, c.** Two examples of binned EDCs from the positions marked by the blue dashed and pink dotted lines in panel a. The fitting algorithm first subtracts a Shirley background, then fits two components built as products of a Lorentzian line shape and the Fermi distribution, subsequently convolved with a Gaussian profile. **d.** The optimized center position of the low-energy fitted component (blue line shape in panels b and c) against the real-space axis of extraction of the binned EDCs. A value close to the Fermi level is the fingerprint of the flat band, while larger negative values correspond to more and more dispersing bands.

The present study reports on the realization of the fragile near-MA configuration in exposed TBG by stacking iso-oriented graphene crystals grown via CVD. In addition to the observation of a moiré pattern by STM and an ARPES signal fully compatible with those achieved with exfoliated graphene flakes in the literature, a spatial analysis is carried out to further characterize the extension of the largest near-MA domain obtained of about 84 μm^2^. The observation and quantitative characterization of a clean and structurally coherent near-MA domain underline the maturity reached by our fabrication protocol and provides compelling evidence that scalable CVD graphene is capable of hosting the fragile flat-band electronic structure previously accessible only in exfoliated systems. Addressing the fabrication of transport devices suitable for investigating correlated electronic phases will, however, require overcoming additional challenges associated with the integration of contacts and gates. Considering our results and recent advancements in the fabrication of CVD-based devices^32,34,35^ and TBG,^36,40^ this objective appears to be within reach. This work thus marks a significant step toward enabling future investigations of correlated quantum states in large-area, synthetically grown graphene.

## Supplementary Material



## Data Availability

Data presented in this article, along with nonproprietary software employed to process and plot it, are available at the following link: https://doi.org/10.48557/MY2BCP.

## Notes

Materials and Methods section; unsupervised machine learning-enabled procedure to automatically detect domains in the sample; detailed procedure for evaluating the near-MA configuration relaxation length; continuum-model calculation, band unfolding, and ARPES spectral weight in twisted bilayer graphene; Supplementary Table S1; Supplementary Figures S1–S7 (PDF)

## #

ICFO - Institut de Ciències Fotòniques, The Barcelona Institute of Science and Technology, Castelldefels (Barcelona), Spain
