# Supplementary material for: Direct Observation of Flat Bands in Near-Magic-Angle Twisted Bilayer CVD Graphene
Source: Nano Lett. 2026 Jul 27;26(30):9759–65. doi: 10.1021/acs.nanolett.6c01400 (PMC13450434; doi:10.1021/acs.nanolett.6c01400)
Supplement: Supplementary file 1 [file nl6c01400_si_001.pdf]

SUPPORTING INFORMATION FOR

Direct observation of flat bands in near-magic-angle twisted bilayer CVD graphene

*Gianluigi Baiardi[1,2], Alex Boschi[1], Giulia Piccinini[1,2 †], Vaidotas Mišeikis[1], Lorenzo Cavicchi[3], Aaron Bostwick[4], Chris Jozwiak[4], Eli Rotenberg[4], Kenji Watanabe[5], Takashi Taniguchi[6], Marco Polini[7], Fabio Beltram[8], Antonio Rossi[1 *], Stiven Forti[1], Sergio Pezzini[8], Camilla Coletti[1 *]*

[1] Center for Nanotechnology Innovation @NEST, Istituto Italiano di Tecnologia, P.za San Silvestro 12, 56127 Pisa PI, Italy.

[2] NEST laboratory, Scuola Normale Superiore, P.za San Silvestro 12, 56127 Pisa PI, Italy.

[3] Scuola Normale Superiore, P.za dei Cavalieri 7, 56126 Pisa PI, Italy.

[4] Advanced Light Source, Lawrence Berkeley National Laboratory, 6 Cyclotron Rd, Berkeley, CA 94720, USA.

[5] Research Center for Electronic and Optical Materials, National Institute for Materials Science, 1-1 Namiki, Tsukuba, Ibaraki 305-0044, Japan.

[6] Research Center for Materials Nanoarchitectonics, National Institute for Materials Science, 1-1 Namiki, Tsukuba, Ibaraki 305-0044, Japan.

[7] Physics department, Università di Pisa, Largo Bruno Pontecorvo 3, 56127 Pisa PI, Italy.

[8] NEST laboratory, Istituto Nanoscienze-CNR and Scuola Normale Superiore, P.za San Silvestro

12, 56127 Pisa PI, Italy.

Materials and methods

Graphene single crystals were epitaxially grown on electropolished and annealed copper foil via low-pressure CVD[1] (in a BM® reactor by Aixtron), and subsequently semi-dry-transferred with the aid of a polymeric stack (prepared with poly(propylene carbonate), PPC, and polymethyl methacrylate, PMMA)[2] on a silicon wafer substrate (with 285-nm thermal oxide) for characterization and selection.

Hexagonal boron nitride (hBN) was exfoliated via a parent/child-tape procedure[3] (Magic tape® by 3M) and released on analogous Si/SiO_2 chips after a short annealing on a hotplate in air (90°C for 1 minute) to increase the release yield.

The selection of graphene crystals was performed by optical microscopy and Raman spectroscopy (employing a 532-nm green laser and an InVia® microscope by Renishaw), both to ensure their cleanness from PMMA residuals and to avoid defective and strained crystals. Two distinct nearby crystals, grown on the same Cu grain and thus sharing the same crystalline orientation, were chosen as original monolayers for the assembly of MATBG. A single hBN flake featuring a flat, uncracked, glue-free area of several tens of micrometers in lateral size was selected by optical microscopy and atomic force microscopy (AFM, with a Dimension Icon® setup by Bruker).

The exposed TBG sample was fabricated via a modified version[4] of the pick-and-flip method originally developed by Wong *et al.*,[5] which incorporates the dry-transfer technique enabled by a polycarbonate/polydimethylsiloxane (PC/PDMS) stamp placed on a glass slide.[6] In this modified procedure, a polyvinyl alcohol (PVA) membrane is deposited on top of the standard stamp and is used to sequentially pick all layers up in bottom-up order, i.e. hBN, bottom graphene, and top graphene; the heterostructure is subsequently brought into contact with a second PC/PDMS stamp (PC by Sigma Aldrich, PDMS Sylgard 184), so that dissolving the PVA film in a water drop allows to flip the sample, which is thermally released on a clean substrate following Purdie *et al.*[6] All involved steps were performed with a custom-built transfer setup, and a twist angle of 1.2° was targeted to compensate for the tendency of TBG to relax towards the Bernal stacking. The final

substrate was a silicon carbide chip, chosen both for the atomic flatness of the terraces exposed on the surface after etching, and as part of an attempt to mitigate the photodoping effect previously witnessed at the synchrotron on samples deposited on Si/SiO_2 wafers.

The exposed TBG was then characterized by all aforementioned techniques, identifying the most promising areas as those featuring a contribution of the strain soliton region (AB-to-BA stacking domain boundary) to the Raman 2D peak at least as high as that of the Bernal regions (see Fig. S1), as suggested by Barbosa *et al.*[7] Cr/Au contacts were evaporated on TBG for electrical grounding. The regions of interest were eventually broomed with prolonged AFM scans in contact mode (with an afm+® setup by Anasys Instruments) to further clean them from PC and lithography resist residuals and improve the signal-to-noise ratio during both scanning tunneling microscopy (STM) and nano-ARPES investigations.

STM imaging was accomplished in-house (LT-STM by Scienta Omicron) at room temperature to confirm the near-magic-angle configuration in the regions of interest by direct observation of the moiré pattern. Nano-ARPES measurements were carried out at the MAESTRO beamline of the Advanced Light Source (ALS) at Lawrence Berkeley National Laboratory (LBNL), California, US. Long-exposure snapshots of the TBG electronic dispersion along k_x, reciprocal-space maps of the full 2D dispersion, and hyperspectral maps with up to 1-μm^2 resolution were all acquired at energies of 27 and 95 eV, respectively with a pass energy of 30 and 50 eV, enabling the spectroscopic characterization of the presented device and the subsequent discussion. For both STM and nano-ARPES experiments a vacuum annealing was carried out at 150°C overnight.

AFM and STM images were processed with Gwyddion 2.68 standard modules.[8] Raman data were elaborated with Renishaw's WiRE proprietary software and, with respect to the more in-depth analysis reported in the SI, by custom Python codes, available on the linked repository (see Data Availability). ARPES data were analyzed via BEAMLINE 7.0.2 Igor Pro routines, other custom Igor Pro routines, and custom Python codes.

With respect to reproducibility, we wish to note that both the sample discussed here and its counterpart on Si/SiO_2, mentioned in the main text, exhibited near-MA hybridization of the Dirac cones, albeit with different domain sizes. An identical data-processing procedure yields an

estimated largest near-MA domain of approximately 10×5 μm² for the Si/SiO_2-supported sample. Among the three samples that were successfully transferred, assembled, and subsequently characterized by nARPES, two exhibited near-MA electronic structure. Furthermore, the largest electronically defined near-MA domain increased from approximately 10×5 μm² in the Si/SiO_2-supported sample to 84±41 μm² in the sample discussed here.

Unsupervised Machine Learning-enabled procedure to automatically detect domains in the sample

Non-negative Matrix Factorization (NMF) allows to factorize a positively-valued matrix N×M, where the N rows represent the items in a collection, the M columns represent the features of such items, and the matrix elements represent the amount of such features in every item, into a pair of positively-valued matrices N×A and A×M, where the arbitrarily long dimension vanishing with the product represents a number of fundamental patterns of features present in the collection of items, able to reconstruct the original dataset with the least possible error (the algorithm leading to the factorization is indeed a compression algorithm which minimizes the distance with the original N×M matrix). The non-negativity of such matrices opens to the interpretation of the rows of the A×M matrix as the description of such patterns in terms of the features, and of the rows of the N×A matrix as the relative weight of each pattern within each item in the collection.

We applied such framework to identify domains in our sample, via ARPES intensity and within the field of view given by Fig. 3a, by treating the x-y position as the items and the k-E dispersed signal as the features. The N×M matrix was obtained by cropping the wavevector and energy axes to the most relevant portions (60 and 145 values respectively) and flattening the 4D dataset into a 704×8700 two-dimensional matrix. The optimal number A of patterns, here electronic dispersion signatures, can be fine-tuned iteratively but an educated guess is possible based on direct inspection of the dataset. We found that 3 signatures yielded the optimal result. The assignment of items (x-y positions) to clusters, in order to identify and visualize the domains, was done by a Gaussian-Mixture Model, preferred to a simple k-means clustering as it enables a probabilistic assignment that keeps track of the smoothness of the transition between clusters. Again, the optimal number of clusters can be found iteratively, but asking for 4 clusters yielded the most sensible result, where each of the first three clusters directly corresponds to a specific electronic signature, and the fourth contains no relevant content of any signature, due to degraded ARPES signal beyond the edge of the sample (bottom-left) or the lack of the TBG structure (top-right).

Detailed procedure for evaluating the near-MA configuration relaxation length via EDCs

1. Define the direction of interest while imaging the domain via spectral data (Fig. S5a).
2. Extract the k- and E-resolved photoemission data corresponding to the relevant positions. Remember to correct the position of the Fermi level, if not calibrated yet, for the fitting algorithm to yield the correct result.
3. Compute binned EDCs by summing the photoemission intensity over a suitable kx interval.
4. For each EDC fit a Shirley background and a number of components equal to the number of bands visible in the photoemission data. To be physically consistent, the components should be the product of a Lorentzian profile and a Fermi distribution function, subsequently convolved with a same-width Gaussian distribution to account for instrumental uncertainty.
5. If possible, to improve the accuracy of the method, repeat the extraction and fitting along parallel axes across the domain, then average the results position-wise.
6. Display the position (in energy) of the Lorentzian profile vs the real-space coordinate marked by the extraction axis.

Continuum-model calculation, band unfolding, and ARPES spectral weight in twisted bilayer graphene

Twisted bilayer graphene (TBG) was described within the standard single-valley continuum model in the layer-sublattice basis $\{|1A\rangle, |1B\rangle, |2A\rangle, |2B\rangle\}$. The single-particle Hamiltonian was written as[9]

$$\hat{H} = \begin{pmatrix} \hat{H}^{(t)} & \hat{U} \\ \hat{U}^{\dagger} & \hat{H}^{(b)} \end{pmatrix}$$

where $\hat{H}^{(\ell=t,b)}$ is the rotated Dirac Hamiltonian of layer ℓ and U is the moiré interlayer tunneling operator. The intralayer blocks were taken in the low-energy $k \cdot p$ form ($\pm$ sign refers to valley)

$$\hat{H}^{(\ell)} = v_D\left[R_\ell(\theta/2)(\hat{\boldsymbol{p}} \mp \hbar \boldsymbol{K}_\ell)\right]\cdot\left(\pm\sigma_x, -\sigma_y\right),$$

and the interlayer tunneling was expanded in the three leading moiré harmonics,

$$\hat{U} = T_0 + T_1\, exp\left(i\boldsymbol{G}_1\cdot\hat{\boldsymbol{r}}\right) + T_2\, exp\left(i\boldsymbol{G}_2\cdot\hat{\boldsymbol{r}}\right),$$

with T_j parameterized by the intrasublattice and intersublattice hopping amplitudes u_0 and u_1.

The eigenstates were obtained by diagonalizing $\hat{H}$ in a plane-wave basis. For crystal momentum $\boldsymbol{\kappa}$ in the first moiré Brillouin zone (mBZ), the Bloch eigenstates were expanded as

$$\langle \boldsymbol{r} \,|\, \boldsymbol{\kappa},\lambda \rangle = \frac{1}{\sqrt{S}} \sum_{\boldsymbol{G}} u_G(\boldsymbol{\kappa},\lambda)\, exp[i(\boldsymbol{\kappa} + \boldsymbol{G})\cdot \boldsymbol{r}],$$

where $\boldsymbol{G}$ belongs to the moiré reciprocal lattice and $u_{\boldsymbol{G}}(\boldsymbol{\kappa},\lambda)$ is a four-component spinor in layer/sublattice space.

To unfold the moiré bands into the primitive Brillouin zone (pBZ) of graphene, primitive-cell momenta were constructed from a given moiré momentum $\boldsymbol{\kappa}$ according to[10]

$$\boldsymbol{k}_i = \boldsymbol{\kappa} + \boldsymbol{G}_i,$$

where $\boldsymbol{G}_i$ runs over the moiré reciprocal lattice vectors that map the state back into the pBZ. The unfolded spectral weight was defined as the projection of the supercell eigenstate onto the primitive-cell Bloch subspace,

$$P_{\boldsymbol{\kappa}\lambda}(\boldsymbol{k}_i) = \sum_{\nu} \left|\langle \boldsymbol{k}_i,\nu \,|\, \boldsymbol{\kappa},\lambda\rangle\right|^2 .$$

Using the plane-wave decomposition above, this weight can be evaluated directly from the moiré eigenvectors as

$$P_{\boldsymbol{\kappa}\lambda}(\boldsymbol{k}_i) = \sum_{\boldsymbol{g}\in pRL} |u_{\boldsymbol{g}+\boldsymbol{G}_i}(\boldsymbol{\kappa},\lambda)|^2 \ ,$$

where the norm includes the sum over the internal layer and sublattice components.

The unfolded spectral function was then computed as[10,11]

$$A(\boldsymbol{k}_i,E) = \sum_{\lambda} P_{\boldsymbol{\kappa}\lambda}(\boldsymbol{k}_i)\delta(E-\varepsilon_{\boldsymbol{\kappa}\lambda}) \, .$$

In the numerical implementation, the Dirac delta was replaced by a finite broadening. A Lorentzian form was used,

$$\delta(E-\varepsilon_{\boldsymbol{\kappa}\lambda}) \rightarrow \frac{\eta}{\pi[(E-\varepsilon_{\boldsymbol{\kappa}\lambda})^2+\eta^2]} \ ,$$

with η the chosen energy-resolution parameter.

Finally, to model ARPES spectra, a free-electron final-state approximation was adopted, and a layer interference factor was included explicitly. The resulting intensity was written as[12]

$$A(\boldsymbol{k}_i,E) = \sum_{\lambda} \sum_{\boldsymbol{g}\in pRL} \left| \sum_{\ell=t,b} \sum_{\alpha=A,B} u_{\alpha\ell,\boldsymbol{g}+\boldsymbol{G}_i}(\boldsymbol{\kappa},\lambda) \, e^{ik_z d_\ell} \right|^2 \delta(E-\varepsilon_{\boldsymbol{\kappa}\lambda}) \, .$$

Here d_ℓ is the vertical position of layer ℓ, taken symmetrically as $d\_t,b = \pm d/2$ with $d \approx 0.34 \, nm$. The out-of-plane photoelectron momentum was evaluated as[12]

$$k_z = \sqrt{\frac{2m}{\hbar^2}\left[\hbar\omega_{ph} - W + \varepsilon_{\boldsymbol{\kappa}\lambda}\right] - |\boldsymbol{k}_i|^2},$$

where $\hbar\omega_{ph}$ is the photon energy, W is the work function, and $\boldsymbol{k}_i$ is the unfolded in-plane crystal momentum. This form captures the attenuation and phase interference between photoelectrons emitted from the two graphene layers.

Supplementary table

	(near-)MA domain size (μm^2)	EDC linewidth (meV)	Flat Band dispersion** (meV)	Moiré gap (meV)	MDC linewidths ($Å^{-1}$)	Background-to-flat-band ratio
Lisi et al., 2021	~50 *		30±15			
Jiang et al., 2023	~50 *	70		140		
Li et al., 2024	~4 *		11±10			
Chen et al., 2024				150±15		
This work	84±41	~118	~24	~155	0.008 – 0.080	0.877

* Very rough estimate of the domain size based on available spectral mapping images.

** Obtained by tracking the position of the maximum intensity across the k-space, not by estimating the linewidth of the low-energy band.

Table S1 – Comparison between quantitative features of the electronic dispersion characterized in this work and published values for analogous near-MA and MA TBG systems.
We extracted and fitted energy dispersion curves (EDCs) at the m point and momentum dispersion curves (MDCs) at -0.5 eV from Fig. 2b. Details on the fitting are reported in Fig. S4. Being the moiré bands folding very sensitive to the twist angle, we observe that the size and depth of the gaps can be related to the average twist within the probed area, while the sharpness of the feature relates to the temperature and the homogeneity in the twist. As such, our domain shows a little higher disorder compared to analogous samples crafted with exfoliated flakes. Conversely, from the analysis of MDCs at -0.5 eV we report sharp bands, with FWHM values reaching 0.008 $Å^{-1}$. This could be explained by a more homogeneous overall orientation of the whole bilayer within the spot size of nARPES, leading to sharper dispersive bands as the Dirac cones do not shift in the reciprocal space.

Supplementary figures

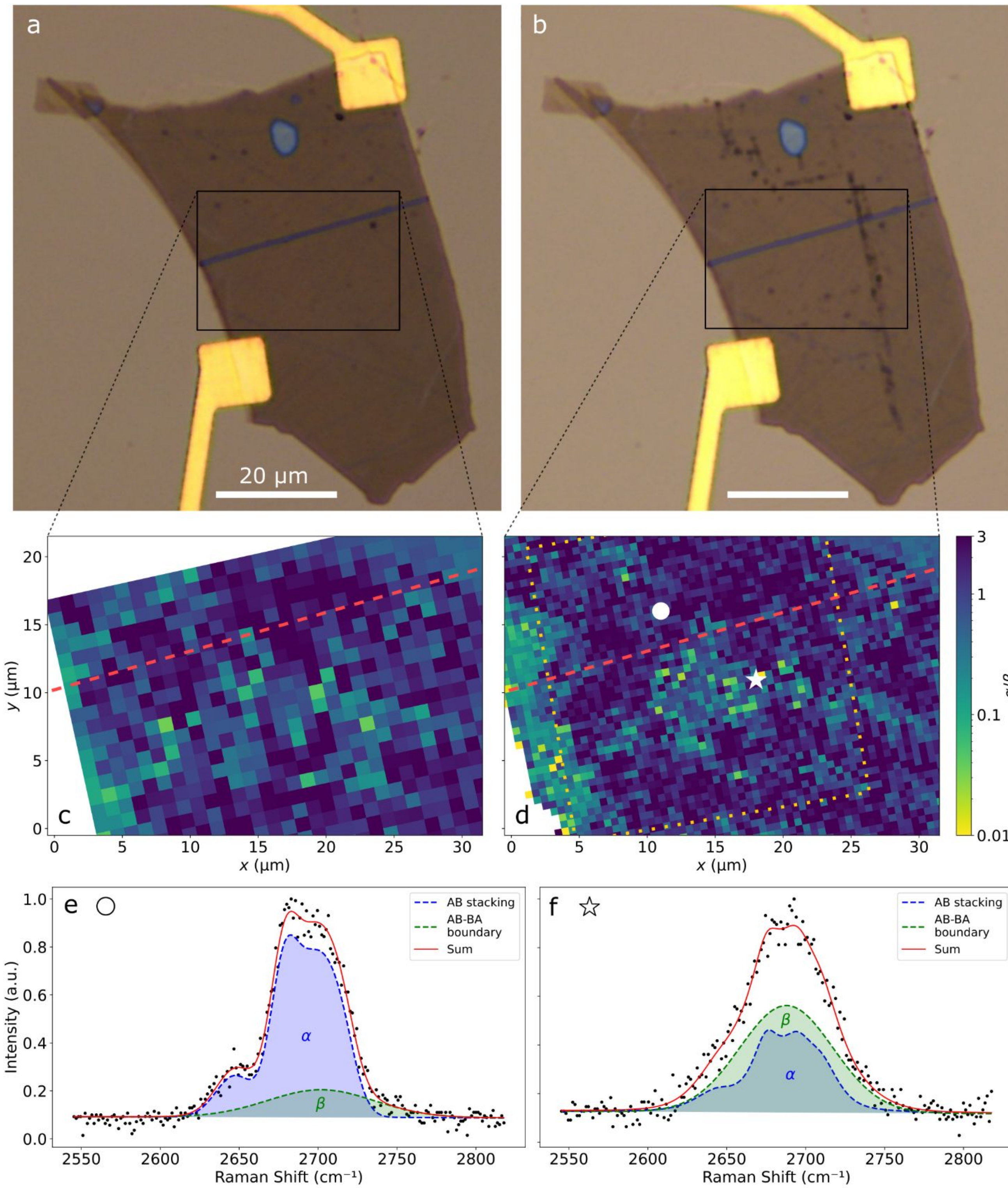

Figure S1 – **a, b** Optical images of the device before and after AFM-assisted brooming, respectively. The scalebar is the same for both panels. **c, d** Raman-derived maps framed in the same area of the ARPES acquisition displayed in Fig. 3a, the different graphical items having the same meaning. The map is built by computing the α/β ratio between the contributions of the Bernal-stacked areas and the strain soliton regions (AB-BA boundaries) to the 2D peak of TBG. Since the two areas in the moiré superlattice are present in different relative amount depending on the twist angle, the procedure allows to initially identify promising near-magic-angle domains by considering α/β values below 0.1, from geometric consideration. Experimentally, the ratio has been reported to saturate to 0.01 immediately above the magic angle[7], a situation which, however, almost never occurs in our sample. On the other end of the spectrum, values of 1 and above strongly point to Bernal-relaxed domains. The method is a more robust alternative to the simple search for areas maximizing the FWHM of the 2D peak by fitting a single Voigt lineshape to an otherwise complex envelope. **e, f** Two examples of fitting of a strain soliton-associated single Voigt and a Bernal-like contribution (four relatively constrained peaks, calibrated on a Bernal bilayer reference sample employing the same laser and spectrometer) to the 2D-peak spectral range of the Raman map acquired on the broomed sample. The data comes from locations marked by the circle and star in panel d, which are the same as in Fig 3. The α/β ratio yielded by the fitting procedure in each point produces the colormap in panel d, where the assignment to Bernal or near-MA configuration is consistent with ARPES data.

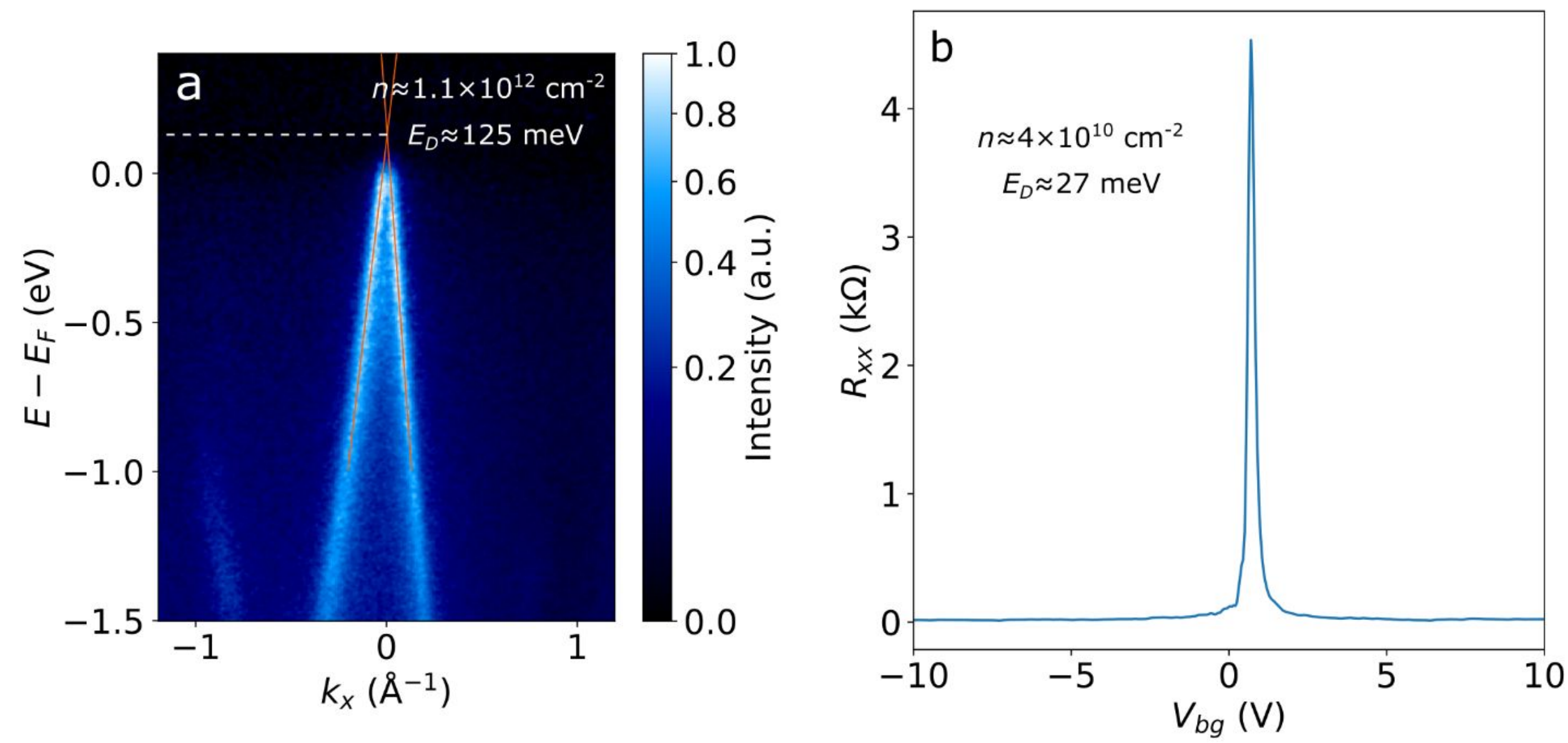

Figure S2 – **a** ARPES signal acquired in a 30°-TBG region of the device in the main text. The snapshot is taken through the Dirac point of the lower layer (part of the Dirac cone belonging to the upper, twisted layer is visible in the bottom-left corner). The graphene appears p-doped of about 125 meV, corresponding to a carrier density of ~1.1×10^{12} cm^{-2}. This value is influenced by charge redistribution between the two graphene layers. Following Piccinini *et al.*,[13] we consider capacitive coupling between the two graphene layers and induction of charge from the underlying hBN/SiC substrate, obtaining a carrier concentration of ~0.8×10^{12} cm^{-2} in the upper layer (note that these estimates are an upper bound: if some of the charge is induced from the top surface, then the graphene would acquire less carriers). Therefore, the total carrier concentration is (at most) ~1.9×10^{12} cm^{-2}, which is smaller than the doping required to completely fill with holes the flat band of MATBG, approximately equal to 3×10^{12} cm^{-2}. **b** Longitudinal resistance of CVD-grown graphene monolayer on hBN, prepared via the same protocols employed for the device in the main text. The back gate (bg) is the degenerately doped Si underneath the thermal oxide; data were acquired at a temperature of 2 K. The original material is confirmed to be minimally p-doped (carrier density approximately equal to 4×10^{10} cm^{-2}, corresponding to a deviation in the Fermi level of about 27 meV from perfect neutrality), hence the attribution of the extensive doping observed in photoemission to a synchrotron-mediated effect. Note that the doping of graphene devices is known not to vary significantly from room temperature (where the ARPES experiment is performed) to cryogenic temperatures (where we perform this reference transport measurement). The estimated mobility at 2 K of this CVD-based single-layer device is ~4×10^{5} cm^2 V^{-1} s^{-1}, with a n* peak-broadening lower than 10^{10} cm^{-2}.

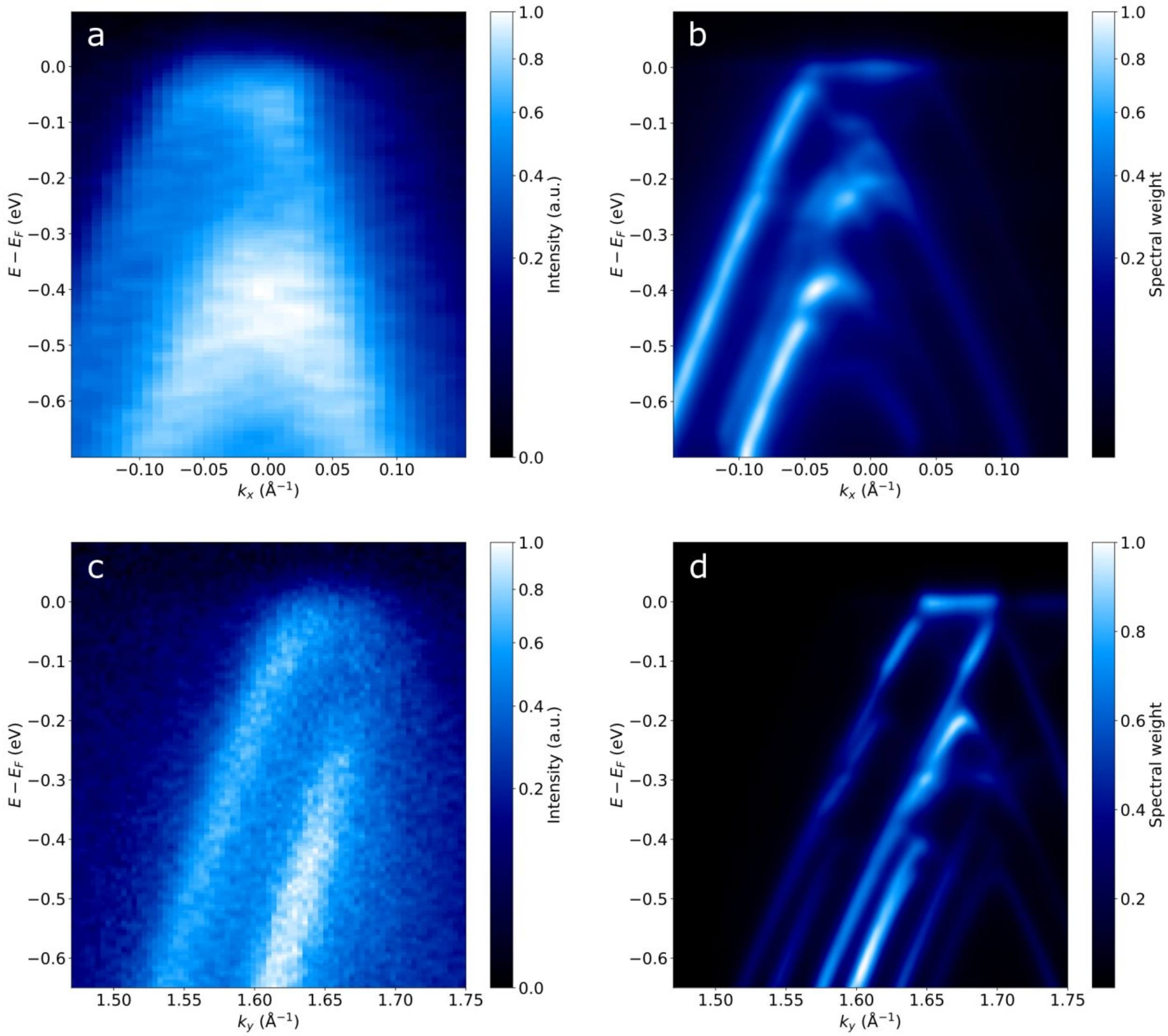

Figure S3 – Comparison between the band dispersion observed in the sample and the electronic structure of MATBG computed with the Bistritzer-MacDonald model. The main morphological features are shared between the experimental data and the calculation, while differences are due to the thermal-induced noise, the potential inhomogeneities in twist angle within the beam spot size, and the slightly different direction of the cut. **a** nARPES signal acquired within the near-MA domain, along the κmκ' axis, slightly off the Dirac points (same data of Fig. 3b in the main text). **b** Spectral weight computed at 1.09°, along the κmκ' axis, with fixed valley index. **c** nARPES signal acquired within the near-MA domain, along the mγm' axis (same data of Fig. 2b in the main text). **b** Spectral weight computed at 1.09°, along the mγm' axis, with fixed valley index.

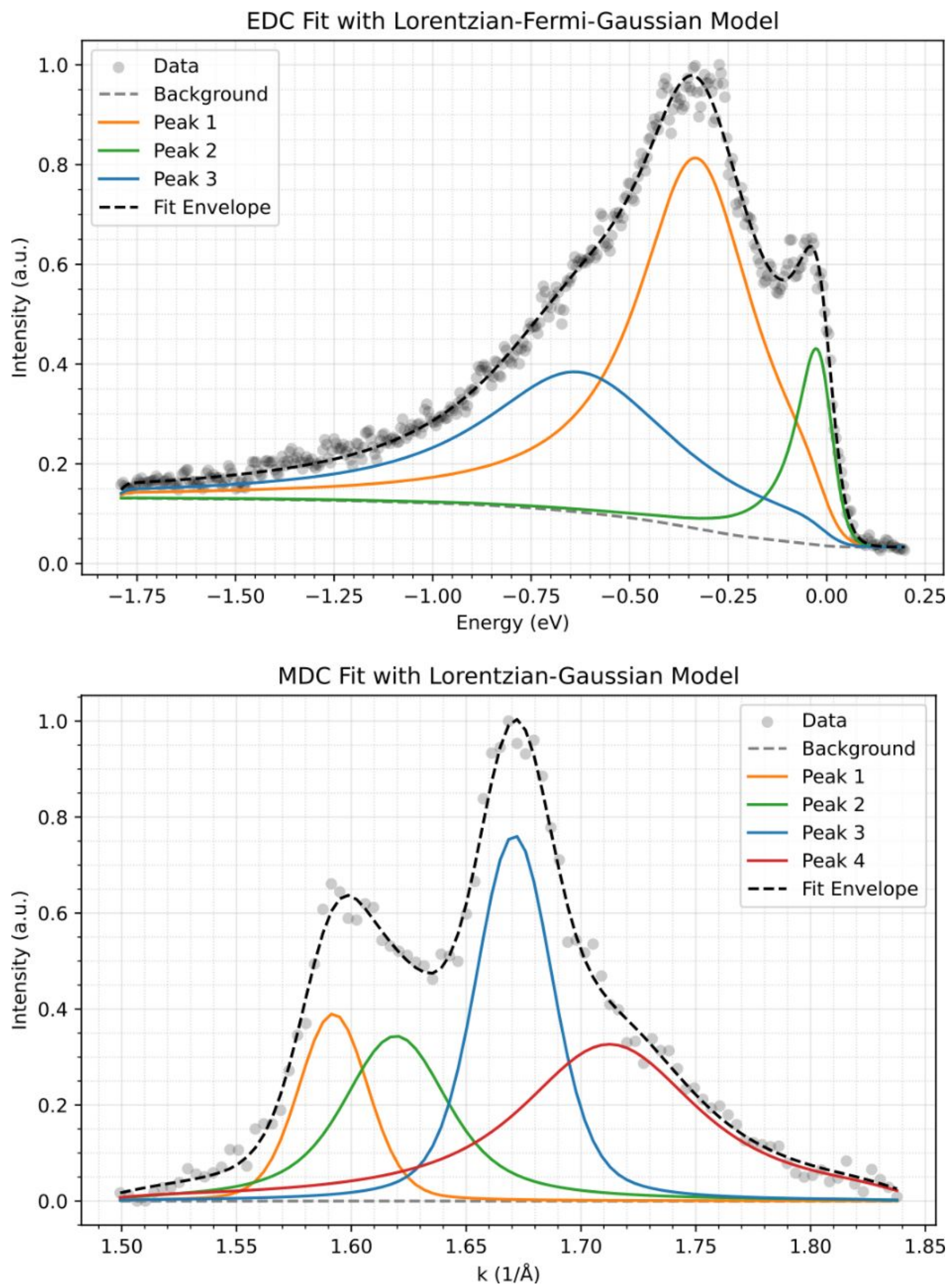

Figure S4 – (**top**) Fit of a minimally binned EDC extracted at the m point of the moiré mini Brillouin zone (from data in Fig. 2b). The Gaussian broadening of the experimental setup, including the thermal contribution, was calibrated by fitting the Fermi edge of p-doped graphene in Fig. S2a and constrained to the value of 14.69 meV (total energy resolution of ~34 meV). The simplest model allowing to reproduce the acquired signal includes three Voigt lineshapes built as described previously in this document, i.e. as product of a Lorentzian profile and a Fermi distribution function, subsequently convolved with a same-width Gaussian distribution. (**bottom**) Fit of a minimally binned EDC extracted at -0.5 eV from data in Fig. 2b. The model is similar to the previous one, excluding the Fermi-Dirac distribution. A minimum number of four components is necessary. The right-hand side of the spectrum is sensibly less intense and resolvable due to dark corridor effects beyond the K point of single graphene layers.

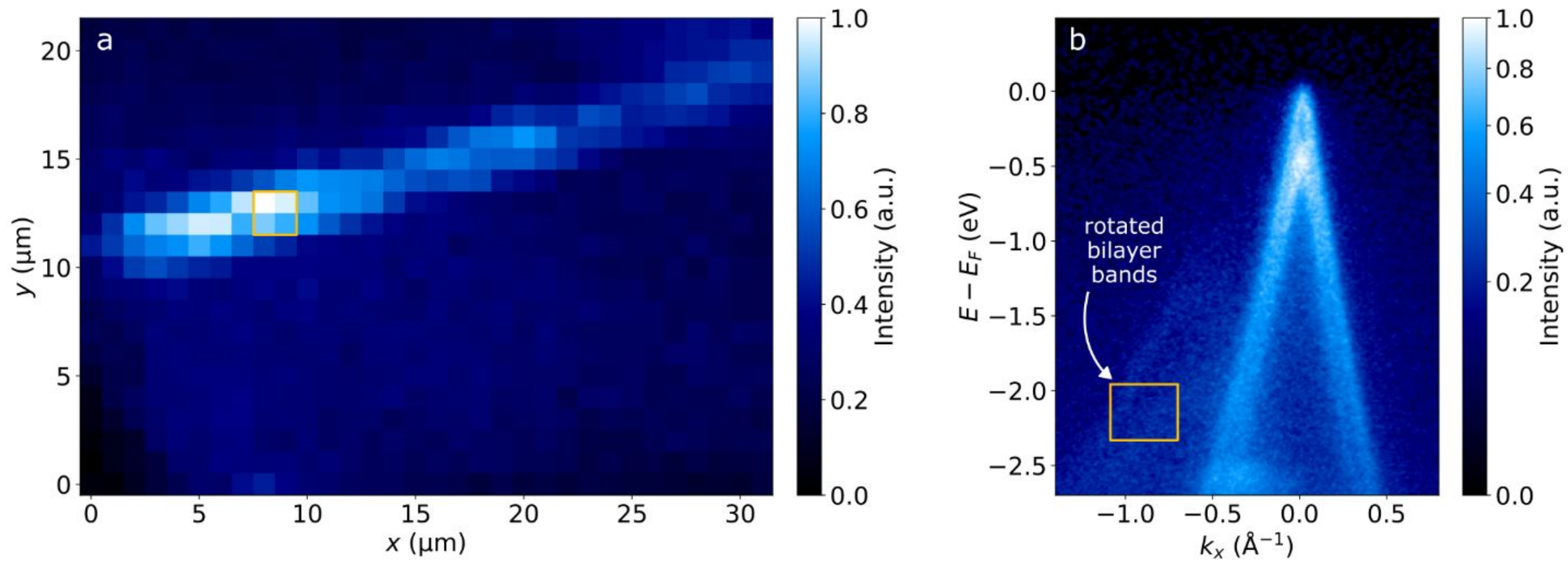

Figure S5 – Study of the TBG relaxation induced by a wrinkle in the underlying hBN flake. **a** Imaging of the wrinkle obtained *via* the ARPES signal, the field of view is the same as that in Fig. 3a. By integrating the signal from a portion of the momentum and energy axes (orange box in (b)), it is possible to image in real space the structure emitting the signal itself. **b** k_x cut of the band dispersion obtained by integration of the signal in the orange box in (a). To the left of the brighter Bernal bilayer dispersion, it is possible to see the faint signature of another Bernal bilayer dispersion cut off the K point, a circumstance occurring when the whole bilayer orientation changes abruptly on a scale below the resolution limit of the technique and both “domains” overlap their signals in the ARPES snapshot. The discontinuity in the bilayer orientation acts as a strong stress on the crystalline order of both layers, giving rise to deformations that lead to the overall relaxation of the near-MA configuration to AB stacking. Note that the TBG appears to have already relaxed to Bernal stacking even before reaching the precise wrinkle position, as panel (b) was obtained by integration over an area of the sample not yet beyond the wrinkle (box in panel (a)): this suggests that the relaxation of the twist angle is not punctual but rather distributed on a micrometric scale according to a mechanical equilibrium of stress and strain. The high-energy feature below -2.5 eV belongs to the valence band of the misaligned hBN flake.

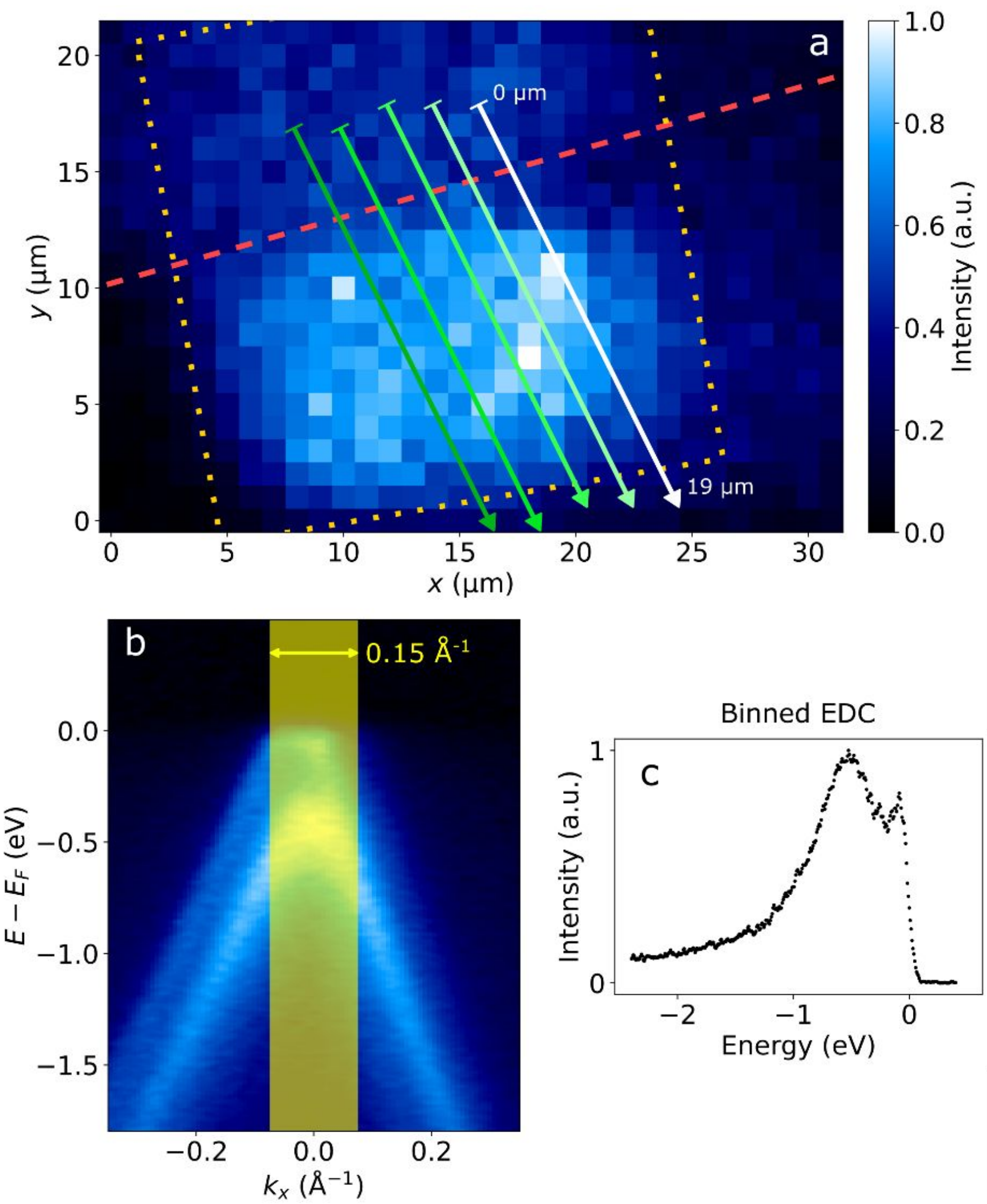

Figure S6 – Extraction of position-resolved k_x-binned EDCs across the near-MA domain. **a** The white arrow marks the direction of extraction of the first series of EDCs, displayed in Fig. 4 in the main text. **b** Employing the data points closest to the chosen axis, EDCs are computed via the binning interval displayed in yellow. **c** Example of a binned EDC, ready for the fitting of its components.

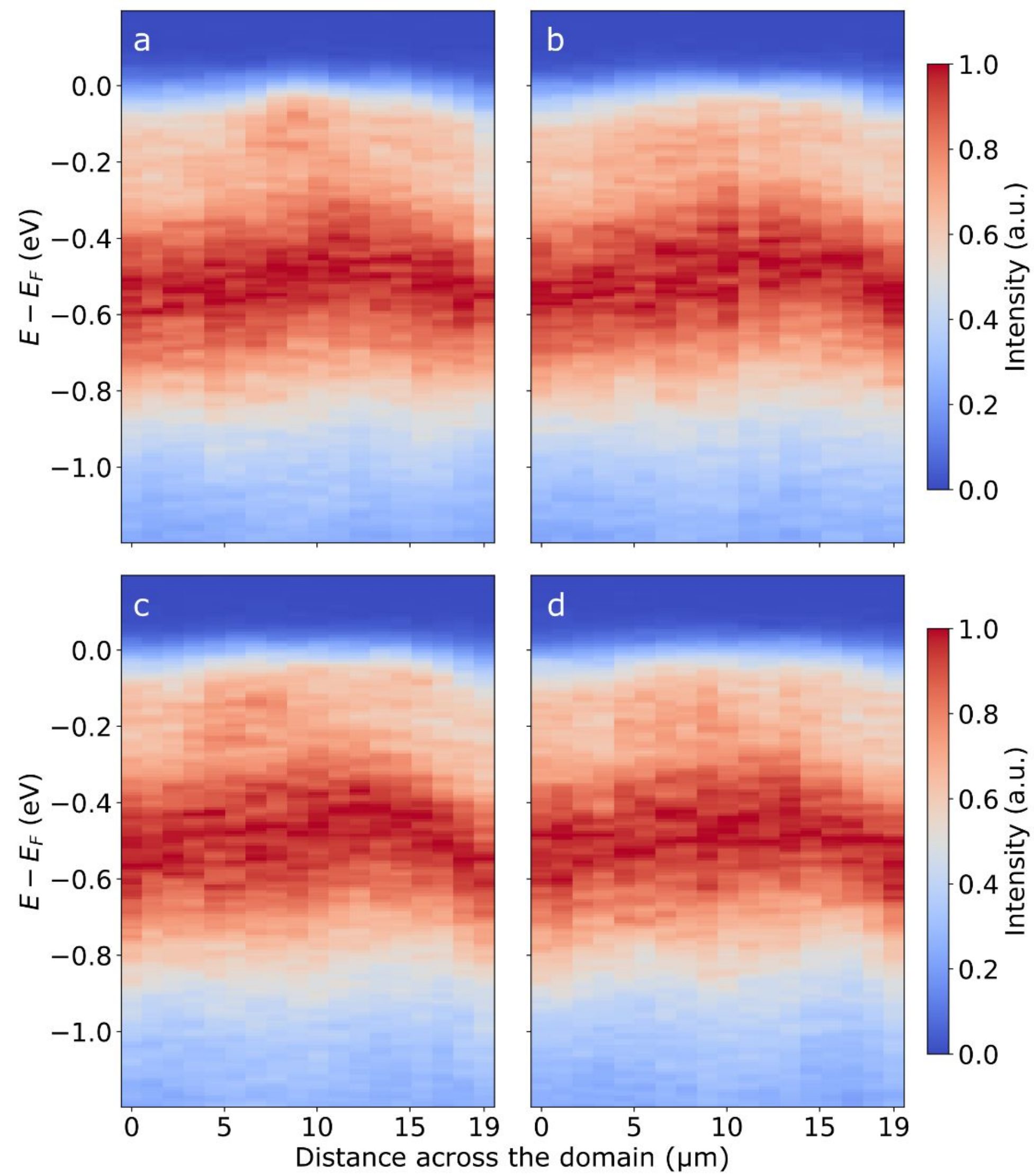

Figure S7 – The other four binned EDC series extracted across the near-magic-angle domain parallelly to the green arrow in Fig. 3a. The morphology of the signal is analogous to that of the first series reported in Fig. 4a.

REFERENCES

(1) Miseikis, V.; Bianco, F.; David, J.; Gemmi, M.; Pellegrini, V.; Romagnoli, M.; Coletti, C. Deterministic Patterned Growth of High-Mobility Large-Crystal Graphene: A Path towards Wafer Scale Integration. *2D Mater.* **2017**, *4* (2), 021004. https://doi.org/10.1088/2053-1583/aa5481.

(2) Giambra, M. A.; Mišeikis, V.; Pezzini, S.; Marconi, S.; Montanaro, A.; Fabbri, F.; Sorianello, V.; Ferrari, A. C.; Coletti, C.; Romagnoli, M. Wafer-Scale Integration of Graphene-Based Photonic Devices. *ACS Nano* **2021**, *15* (2), 3171–3187. https://doi.org/10.1021/acsnano.0c09758.

(3) Cohen, L. A.; Samuelson, N. L.; Wang, T.; Klocke, K.; Reeves, C. C.; Taniguchi, T.; Watanabe, K.; Vijay, S.; Zaletel, M. P.; Young, A. F. Nanoscale Electrostatic Control in Ultraclean van Der Waals Heterostructures by Local Anodic Oxidation of Graphite Gates. *Nat. Phys.* **2023**, *19* (10), 1502–1508. https://doi.org/10.1038/s41567-023-02114-3.

(4) Montanaro, A.; Piccinini, G.; Mišeikis, V.; Sorianello, V.; Giambra, M. A.; Soresi, S.; Giorgi, L.; D'Errico, A.; Watanabe, K.; Taniguchi, T.; Pezzini, S.; Coletti, C.; Romagnoli, M. Sub-THz Wireless Transmission Based on Graphene-Integrated Optoelectronic Mixer. *Nat. Commun.* **2023**, *14* (1), 6471. https://doi.org/10.1038/s41467-023-42194-6.

(5) Wong, D.; Nuckolls, K. P.; Oh, M.; Lian, B.; Xie, Y.; Jeon, S.; Watanabe, K.; Taniguchi, T.; Bernevig, B. A.; Yazdani, A. Cascade of Electronic Transitions in Magic-Angle Twisted Bilayer Graphene. *Nature* **2020**, *582* (7811), 198–202. https://doi.org/10.1038/s41586-020-2339-0.

(6) Purdie, D. G.; Pugno, N. M.; Taniguchi, T.; Watanabe, K.; Ferrari, A. C.; Lombardo, A. Cleaning Interfaces in Layered Materials Heterostructures. *Nat. Commun.* **2018**, *9* (1), 5387. https://doi.org/10.1038/s41467-018-07558-3.

(7) Barbosa, T. C.; Gadelha, A. C.; Ohlberg, D. A. A.; Watanabe, K.; Taniguchi, T.; Medeiros-Ribeiro, G.; Jorio, A.; Campos, L. C. Raman Spectra of Twisted Bilayer Graphene Close to the Magic Angle. *2D Mater.* **2022**, *9* (2), 025007. https://doi.org/10.1088/2053-1583/ac4af9.

(8) Nečas, D.; Klapetek, P. Gwyddion: An Open-Source Software for SPM Data Analysis. *Open Phys.* **2012**, *10* (1), 181–188. https://doi.org/10.2478/s11534-011-0096-2.

(9) Bistritzer, R.; MacDonald, A. H. Moiré Bands in Twisted Double-Layer Graphene. *Proc. Natl. Acad. Sci.* **2011**, *108* (30), 12233–12237. https://doi.org/10.1073/pnas.1108174108.

(10) Popescu, V.; Zunger, A. Extracting E versus $\stackrel{P\vec}{k}$ Effective Band Structure from Supercell Calculations on Alloys and Impurities. *Phys. Rev. B* **2012**, *85* (8), 085201. https://doi.org/10.1103/PhysRevB.85.085201.

(11) Voit, J.; Perfetti, L.; Zwick, F.; Berger, H.; Margaritondo, G.; Grüner, G.; Höchst, H.; Grioni, M. Electronic Structure of Solids with Competing Periodic Potentials. *Science* **2000**, *290* (5491), 501–503. https://doi.org/10.1126/science.290.5491.501.

(12) Nunn, J. E.; McEllistrim, A.; Weston, A.; Garcia-Ruiz, A.; Watson, M. D.; Mucha-Kruczynski, M.; Cacho, C.; Gorbachev, R. V.; Fal'ko, V. I.; Wilson, N. R. ARPES Signatures

of Few-Layer Twistronic Graphenes. *Nano Lett.* **2023**, *23* (11), 5201–5208. https://doi.org/10.1021/acs.nanolett.3c01173.
(13) Piccinini, G.; Mišeikis, V.; Watanabe, K.; Taniguchi, T.; Coletti, C.; Pezzini, S. Parallel Transport and Layer-Resolved Thermodynamic Measurements in Twisted Bilayer Graphene. *Phys. Rev. B* **2021**, *104* (24), L241410. https://doi.org/10.1103/PhysRevB.104.L241410.
